# Everybody's Page

**Published:** 1893-02-25

**Authors:** 


### Everybody's Page.

We talk a great deal about our medical officers of health and the prevention of disease, but the former would succeed better in the latter if they had more power given to them. Scarlet fever would never have assumed its recent proportions if it had been dealt with in England as small-pox is in Ceylon, for instance. In that country the natives consider that dire disease of so little importance that stringent measures are needful, and doctors are authorised to have houses containing cases of infectious disease placarded with a notice of the fact in at least three different languages. In a dwelling which is entirely detached and satisfactory isolation is rendered possible, the placard may be omitted; but where habitations are crowded together he has no option in the matter.

One must cut one's coat after all, according to one's cloth and Professor Morse's novel scheme for house warming may suit the climate of Massachusetts, it certainly would not suit ours. To put his theory into effect, Professor Morse has built a model house at Salem, which is so constructed that all its apartments face due south, the corridors alone looking north. The frontage of the building is of glass, with reflectors to "repeat" the power of the sun. This, the professor believes, is the most wholesome form of house heating extant, and, indeed, we do not feel inclined to contradict him on this head, but in countries of, say, uncertain sunshine, this heating would surely be a question more of theory than of fact, and in depending on an exterior source for heat the inmates would be likely to perish in the attempt.

